# Notes and Comments

**Published:** 1896-10

**Authors:** 


### Notes and Comments.[1]

We may learn from the Humblest.—Dr. T. B. Welch says that the man who "knows everything" and "can do anything" is usually a blockhead. The man who is willing to sit at the feet of the humblest, and can learn something from the most unassuming, becomes rich in variety of store, and that store of wealth is sure to be so manipulated as to serve him as wisdom on many an exigency. The true scholar treasures up something valuable from every one and from every phase of life.

Pluck, not Luck.—Luck, as Rev. J. G. Rust truly says, is a foolish doctrine of fate; it is the silliness of superstition; it is the

[1] The assistant editor solicits contributions for this department,—new methods, new remedies and formulas, or any short practical note which may prove of value to the practitioner or student. Address 1718 Walnut Street, Philadelphia.

cynicism of fools, incompetents, and failures. You never hear a real sensible man talking about luck; he knows the philosophy of success too well; he knows the meaning of patience and pains-taking care, of energy and economy.

---

PYORRHŒA ALVEOLARIS.—In listening to Dr. Talbot's paper upon pyorrhœa alveolaris, read before the Academy of Stomatology of Philadelphia, we noted his remark that a general invasion of this disease—that is, attacking all the teeth—"never occurs." This is an error, as such cases frequently occur, and no doubt are observed by many practitioners. The writer has treated more than one case in his own practice where every tooth was affected, and has met several in the college clinics.

---

FUNCTION OF THE VACUUM-CHAMBER.—In a contribution to the *Dental Cosmos*, Dr. Henry Burchard claims that the vacuum-chamber has an *enduring function*, and offers as a proof of this the statement that by temporarily filling the depression or chamber with wax, or by perforating the wall of the chamber, the result, in either case, would be the almost total loss of adhesion.

The doctor is correct where he says filling the chamber with wax would interfere with adhesion, but this is owing to the fact that, as a *result of the chamber*, the soft tissue is drawn down and into it; the wax would therefore press upon this hypertrophied portion, preventing the plate from going to place. As perfect adhesion depends upon perfect adaptation, anything that interferes with the latter tends to destroy the former.

The best evidence of the uselessness of vacuum-chambers (excepting for temporary assistance) is the fact that full upper dentures are continually being successfully placed in the mouth without them.

---

NEW FORMULA FOR LOCAL ANÆSTHETIC.—The formula given below is claimed to be an efficient local anæsthetic, and is original with Dr. W. D. Dalrymple, Ogden, Utah:

℞ Cocaine hydrochlorate, grs. ii;

Carbolic acid, gtts. x;

Glycerin, ʒi;

Listerine, ℥i;

Aqua dest., ad q. s., ℥ii.—M.

